# Viral load detection and management on first line ART in rural Rwanda

**DOI:** 10.1186/s12879-018-3639-y

**Published:** 2019-01-03

**Authors:** Jean de Dieu Ndagijimana Ntwali, Tom Decroo, Muhayimpundu Ribakare, Athanase Kiromera, Placidie Mugwaneza, Sabin Nsanzimana, Lutgarde Lynen

**Affiliations:** 1 Institute of HIV Disease Prevention and Control, Rwanda Biomedical Centre, 17 avenue, Po. Box 7162, Kigali, Rwanda; 2 Department of Clinical Sciences, Institute of Tropical Medicine, Antwerp, Belgium; 3 Research Foundation Flanders, Brussels, Belgium; 4 Institute of Human Virology, Rwanda Program, University of Maryland, Kigali, Rwanda

**Keywords:** Anti-retroviral therapy, viral load, viral suppression, Detectable viral load

## Abstract

**Background:**

To achieve the ambitious 90–90-90 UNAIDS targets, access to routine viral load (VL) is critical. To measure VL, Rwanda has relied on a national reference laboratory for years. In 2014, a VL testing platform was implemented in a rural District in the Northern Province. Here we analyze the uptake of VL testing, identification of risks for detectable VL (≥1000 copies/ml), and the management of patients with a detectable VL.

**Methods:**

A retrospective cohort study of patients who started ART between July 2012 and June 2015 and followed until end December 2016. Using descriptive statistics, we describe the VL cascade, from VL uptake to the start of second-line ART in patients diagnosed with virological failure. We estimate predictors of having a detectable VL using logistic regression.

**Results:**

The uptake of VL testing increased progressively between 2013 and 2016, raising from 25.6% (39/152) in 2013 up to 93.2% (510/547) in 2016.In 2016, 88.5% (*n* = 451) of patients tested, had a suppressed VL. Predictors of having a detectable VL included being male (aOR 2.1; 95%CI 1.12–4.02; *p* = 0.02), being a sex worker (aOR 6.4; 95%CI 1.1–36.0; *p* = 0.04), having a WHO clinical stage IV when starting ART (aOR 8.8; 95%CI 1.8–43.0; *p* < 0.001), having had a previous detectable VL (aOR 7.2; 95%CI 3.5–14.5; p < 0.001), and having had no VL before 2016 (aOR 3.1; 95%CI 1.2–8.1; p = 0.02). Among patients with initial detectable VL between 2013 and 2016, 88% (*n* = 103) had a follow-up VL, of whom 60.2% (*n* = 62) suppressed their VL below 1000 copies/ml. The median time between the initial and follow-up VL was of 12.5 months (IQR: 8.7–19.0). Among patients with confirmed treatment failure, 63.4% (*n* = 26) started second-line ART within the study period.

**Conclusion:**

VL uptake increased after decentralizing VL testing in rural Rwanda. Virological suppression was high. An individualized follow up of patients at risk of non-suppression and a prompt management of patients with detectable VL may help to achieve and sustain the third global UNAIDS target: virological suppression in 90% of patients on ART.

## Background

Despite significant efforts made in the prevention and the fight against HIV, the number of annual new HIV infections remains high [1]. The sub-Saharan Africa region carries the highest burden of the disease, with nearly 70% of all people living with HIV (PLHIV), and 76% of all HIV related deaths coming from this region [2]. In 2015, UNAIDS launched the ambitious 90–90-90 targets: by 2020, 90% of all PLHIV should know their serological status, 90% of all diagnosed PLHIV should receive antiretroviral treatment (ART), and 90% of all PLHIV on ART should have a suppressed viral load (VL) [3]. This corresponds with 73% of all PLHIV having a suppressed VL. By achieving these targets, HIV transmission and HIV related mortality should decrease drastically.

To monitor progress towards achieving these targets, routine VL monitoring is indispensable. However, due to logistical and financial constraints, most sub-Saharan ART programs are unable to provide a regular access to VL testing for most of their patients on ART. A review showed important disparities between seven sub-Saharan Africa countries and revealed a number of countries without adequate capacities to perform VL test to all their patients on ART. The percentage of patients on ART with at least one VL test done ranged from 9% in Tanzania to 91% in South Africa [4].

In the last 20 years, Rwanda has made tremendous progress in the fight against HIV, with a massive increase of HIV counseling and testing services and the decentralization of ART provision [5]. Today, in Rwanda, the first and the second UNAIDS targets are close to be reached: in 2016, an estimated 87% of 220,000 PLHIV knew their status and 80% of PLHIV were on ART [6]. However, achieving and monitoring the third UNAIDS target may be more challenging.

Since 2011, the Rwandan national guideline recommends routine annually VL testing in all patients on ART. However, its implementation has been challenged by the insufficient number of VL testing platforms, especially in the rural areas. In the last three years, the number of VL testing platforms has increased progressively. In addition, health care providers were trained on indications and the importance of having a routine VL [7]. Two studies conducted in Rwanda showed a high variability: virological suppression ranged between 82.1% virological suppression in adult patients on ART for the first study, [8] and 52.2% virological suppression in pregnant women on ART for the second study [9]. Furthermore, the management of patients with detectable VL was not studied.

In most resource-poor countries, VL testing is not decentralized. Hence, samples need to be transported to a central laboratory, which reduces accessibility to the VL testing [10]. We therefore studied the use of routine VL testing in patients on ART in the rural area of Rwanda, after installing a VL testing platform in the District of Musanze. We identified predictors of virological failure and described the management of patients with a detectable VL.

## Methods

### Study design

This is a retrospective cohort study, using routinely collected HIV program data. Data were collected in one public hospital and one health center in the Northern Province of Rwanda.

### General setting

Rwanda is a country with 11.5 million inhabitants, located in the Eastern African region. With 467 people per Km^2^, the country has the highest density of population in Africa. Still, 82% of the population lives in a rural area [11]. Life expectancy at birth is 67 years for women and 64 years for men.

In Rwanda, the HIV epidemic is stable, with a prevalence of 3% in the general population. The prevalence is higher in urban areas (6.3%) than in rural areas (2.4%), and in females (3.6%) than in males (2.2%) [12]. The HIV prevalence is much higher in key populations, such as female sex workers, with a prevalence of 41.4% [13]. The management of PLHIV is decentralized to the level of primary health care, with 96% of all public health facilities (*n* = 545) offering voluntary counselling and testing services (VCT), the provision of ART and prevention of mother to child transmission (PMTCT) services.

### Study setting

Ruhengeri hospital and Musanze health center are two neighboring health facilities, both located in the District of Musanze in the Northern Province of Rwanda. The HIV prevalence in this district is 2.7% [12].

Ruhengeri district hospital is among the first health facilities that offered HIV services in Rwanda. HIV services are available since 2003. In 2016, the hospital counted 1605 patients actively followed up on ART. One medical doctor, four nurses and two social workers are assigned to provide HIV care. Since 2014, a VL testing platform using COBAS Amplipera and COBAS AmpliTaq machines has been available in Ruhengeri hospital. It serves to analyze samples from the hospital and neighboring health facilities. Using EDTA tubes, whole blood samples are sent from the health center to the hospital. A standardized external quality control is conducted twice a year in collaboration with the Center for Diseases Control in Atlanta-USA. The hospital uses OpenMRS software for the daily recording of patient’s data. Each patient has a unique TracNet identification number.

Muhoza health center opened in May 2011. In 2016, the health center provided care for 973 active patients on ART. HIV care is provided by a team of three nurses and one social worker. Samples from the health center are sent to Ruhengeri district hospital for VL testing.

### Study population and period

All patients who were initiated on ART between the 1st of July 2012 and the 30th of June 2015 in both study sites were included in the study. Data were collected between the dates each patient started ART until the date of 31st of December 2016 which is the end of the study period. However, for patients who exited the program before the above mentioned date, we considered the date when those patients were transferred out, lost to follow-up, or reported as dead.

### Data collection and definition of variables

Routine program data were collected from patient charts, ART registers and VL registers, available at both sites. This information was completed with data collected from the OpenMRS database. Collected data comprised socio-demographic and clinical variables.

Socio-demographic variables included age, gender, area of residency, marital status, and current occupation. Clinical variables included the date of ART initiation, ART regimen, VL result, date of VL result, date of second-line ART initiation, treatment outcomes on 31/12/2016 (dead, lost to follow-up, transferred out, active in care).

A patient is defined as ‘active on ART’ if he/she is in care at a given time, and has attended at least a pharmacy refill appointment within the last three-month period.

The patient was ‘lost to follow-up’ (LTFU) if he/she didn’t attend the pharmacy refill appointment for more than three consecutive months.

A patient was considered as ‘transferred out’, if for personal reasons, he decided to continue follow-up in a different health facility and received a transfer form from Ruhengeri hospital or Muhoza health center. Characteristics of patients transferred out were similar to those who remained in care.

We defined a suppressed VL as each VL result less than 1000 copies/ml, and a detectable VL as a VL ≥ 1000 copies/ml. Virological failure was defined as two consecutive VL ≥ 1000 copies/ml, with at least a three-month interval between the initial and the control VL. Retention was calculated as the proportion of active patients among the study population at the end of the study period, excluding those who were transferred out from both the nominator and denominator (as their treatment outcomes could not be ascertained on 31st December 2016).

The data collection was conducted by nurses working in the two study sites and data were encoded in an Excel database. A TracNet identification number was linked to each patient in the study, and the identity of patients was confidential at each step of the data collection.

### Data analysis

Baseline characteristics of patients in our cohort were described using standard descriptive statistics. Univariate analysis was conducted using measures of frequency, median and interquartile range (IQR) for numeric variables and proportions for categorical variables. Multivariate analysis was conducted using logistic regression to establish a predictive model for having a detectable VL in 2016. The final model was retained when the likelihood ratio resulted in a *P* < 0.05.

In patients with a detectable initial VL between July 2012 and June 2015, we calculated the proportion that had a follow-up VL, and the delay between the high initial VL and the follow-up VL. According to the Rwanda national protocol, patients with a VL ≥ 1000 copies/ml should have a follow-up VL within six months. Patients have to be active in care to measure their VL. Therefore, we calculated the proportion of patients with a follow-up VL among those who were active six months after a high initial VL.

Data were collected using Excel 2010 and analyzed using EPI info 3.5.4.

### Ethics

No identifiers were encoded in the study database. This study was approved by the Ethics Committee of Ruhengeri provincial hospital as well as the Institutional Review Board of the Institute of Tropical Medicine of Antwerp, Belgium.

## Results

A total of 775 patients enrolled on ART between July 2012 and June 2015 were included in the study. Most (67%) were female. The median age of patients enrolled on ART was 34 years (IQR: 27–41) (Table 1). Most (61.2%) patients were farmers. Of all patients, 48.1 and 51.9% started ART in the hospital and in the health center, respectively.

**Table 1:** Characteristics of patients enrolled on ART, in Ruhengeri hospital and Musanze health center, 2013–2016

Characteristics	*N*	%
Sex		
Female	519	67.0%
Age, Median (IQR)	34	27–41
0–14	54	7.0%
15–29	206	26.6%
30–49	443	57.2%
50 and above	72	9.3%
Health facility		
Hospital	373	48.1%
Health center	402	51.9%
Area of residency		
< 5 km	523	67.5%
5–10 Km	229	29.5%
> 10 Km	23	3.0%
Marital status		
Married	439	56.6%
Not married	336	43.4%
Current occupation		
Farmer	474	61.2%
Public/ Private servants	207	26.7%
Sex worker	13	1.7%
Unemployed	81	10.5%
Mode of enrollment		
VCT	389	50.2%
PIT	136	17.5%
PMTCT	161	20.8%
Not registered	89	11.5%
WHO Clinical Stage		
1	401	51.7%
2	159	20.5%
3	198	25.5%
4	17	2.2%
CD4 Count at ART initiation		
0–199	115	14.8%
200–500	438	56.5%
500 and above	222	28.6%
Initial ART regimen		
TDF + 3TC + EFV	682	88.0%
Other ART regimen	93	12.0%

ART: antiretroviral therapy; IQR: interquartile range; km: kilometer; N: number; PIT: provider initiated testing; PMTCT: prevention mother to child transmission; VCT: voluntary counselling and testing; WHO: World Health Organization

Of 775 patients, about half were asymptomatic when they started ART (51.7%). The median CD4 count at ART initiation was 364 cells /mm^3^ (IQR: 265–531). At ART initiation, the vast majority of patients (88%) were prescribed TDF + 3TC + EFV combined regimen.

At the end of the study period, 70.6% (*n* = 547) were active on ART; 21.4% (*n* = 166) were transferred out to another health facility, 2.7% (*n* = 21) died and 5.3% (*n* = 41) were lost to follow up. Retention on ART was 95.8% after one year on ART, 93.4 and 92% respectively after two and three years on ART. The median follow-up time for our cohort was 29 months (IQR: 21–41) on ART.

**Fig. 1:**
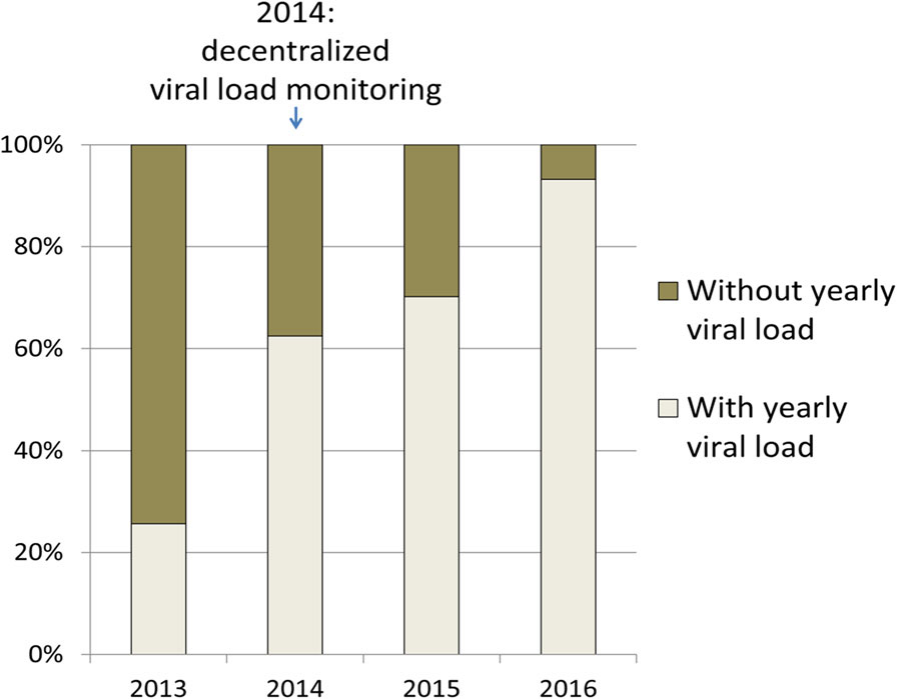
Trend in viral load monitoring in a rural district of Rwanda, 2013–2016

The uptake of VL among patients on ART has been gradually increasing since 2013. (Fig. 1) The proportion of patients active on ART that had a yearly VL increased from 25.6% (39/152) to 62.5% (195/312) between 2013 and 2014 (*p* < 0.001), from 62.5% (195/312) to 70.2% (347/494) between 2014 and 2015 (p < 0.001), and from 70.2% (347/494) to 93.2% (510/547) between 2015 and 2016 (*p* < 0.0001). Yearly VL uptake increased significantly during the four consecutive years, between 2013 and 2016.

**Fig. 2:**
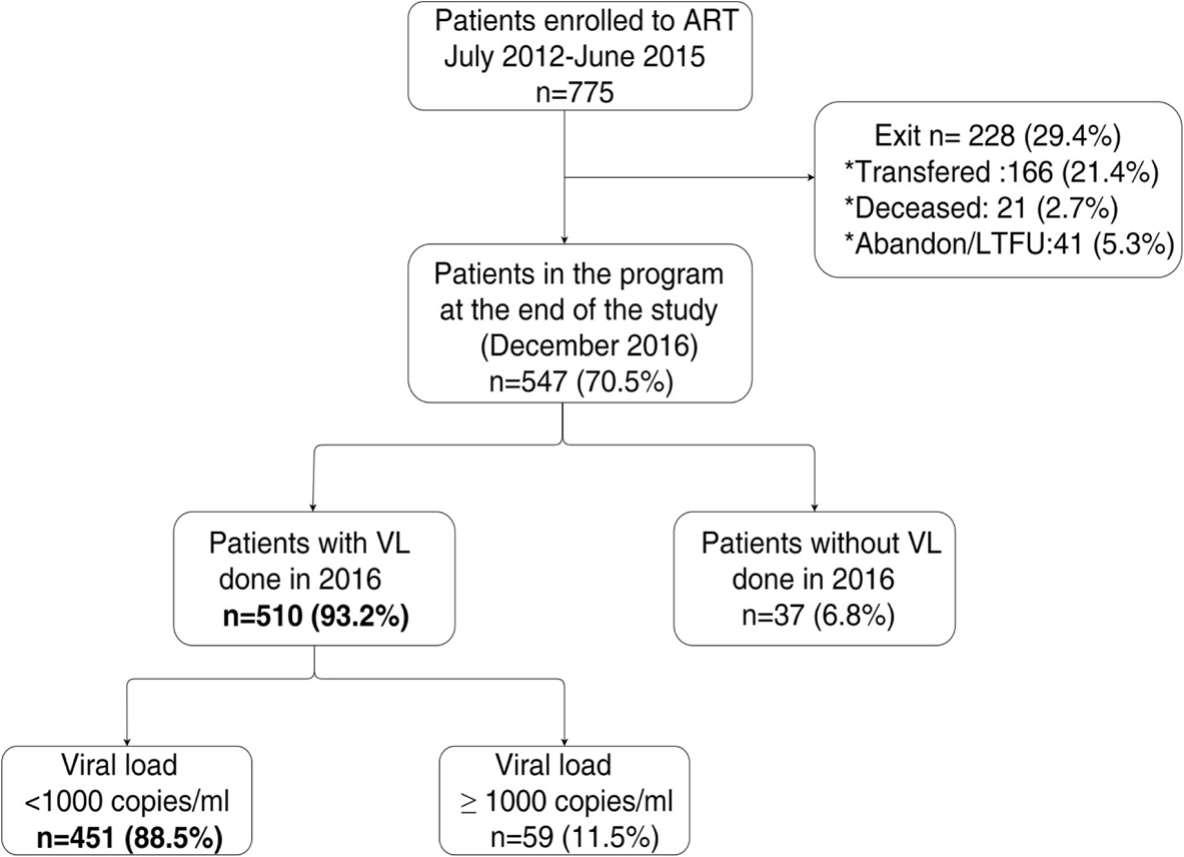
Viral load detection and results in Rwanda: Ruhengeri hospital and Musanze health center, in 2016. ART: antiretroviral therapy; VL: Viral load; LTFU: lost to follow-up; n: number.

Out of 510 patients with a VL result in 2016, 88.5% (*n* = 451) had a suppressed VL (< 1000 copies/ml) and 11.5% (*n* = 59) had a detectable VL (≥ 1000 copies/ml) (Fig. 2). Predictors of having a detectable VL included being male (aOR 2.1; 95%CI 1.12–4.02; *p* = 0.02), being a sex worker (aOR 6.4; 95%CI 1.1–36.0; *p* = 0.04), having a WHO clinical stage IV when starting ART (aOR 8.8; 95%CI 1.8–43.0; *p* < 0.001), having had a detectable VL before 2016 (aOR 7.2; 95%CI 3.5–14.5; p < 0.001), and having had no VL before 2016 (aOR 3.1; 95%CI 1.2–8.1; p = 0.02) (Table 2).

**Table 2:** Patients with a viral load ≥1000 copies/ml, in Ruhengeri hospital and Musanze health center, in Rwanda, in 2016

Characteristics	VL done in 2016	Patients with VL ≥ 1000 copies in 2016				
		*N(%)*	*Crude OR (95%IC)*	*P-value*	*Adjusted OR (95%IC)*	*P-value*
Total	510	59 (11.5)				
Sex						
Female	345	29 (8.4)	1-		1-	
Male	165	30 (18.2)	2.4 (1.4–4.2)	0.001	2.1 (1.1–4.0)	0.02
Age group				0.07		
30–49	304	30 (9.9)	1-		–	
15–29	109	12 (11.0)	1.1 (0.5–2.3)	0.7		
50 et Plus	55	7 (12.7)	1.3 (0.5–3.2)	0.5		
0–14	42	10 (23.8)	2.8 (1.3–6.4)	0.01		
Health facility						
Hospital	240	29 (12.1)	1-		–	
Health center	270	30 (11.1)	0.9 (0.5–1.5)	0.7		
Area of residence				0.9		
< 5 km	348	41 (11.8)	1-		–	
5-10 km	148	16 (10.8)	0.9 (0.5–1.7)	0.8		
> 10 km	14	2 (14.3)	1.2 (0.2–5.8)	0.8		
Marital status						
Married	292	30 (10.3)	1-		–	
Not married	218	29 (13.3)	1.3 (0.8–2.3)	0.3		
Current occupation				0.01		
Farmer	311	30 (9.6)	1-		1-	
Public/private servant	133	15 (11.3)	1.2 (0.6–2.3)	0.6	1.1 (0.5–2.3)	0.8
Unemployed	59	11 (18.6)	2.1 (1.0–4.5)	0.05	1.3 (0.5–3.1)	0.5
Sex worker	7	3 (42.9)	7.0 (1.5–32.8)	0.01	6.4 (1.1–36.0)	0.03
Mode of enrollment				0.3		
VCT	262	33 (12.6)	1-		–	
PIT	85	13 (15.3)	1.2 (0.6–2.5)	0.5		
PMTCT	100	7 (7.0)	0.5 (0.2–1.2)	0.1		
Transferred In	63	6 (9.5)	0.7 (03–1.8)	0.5		
WHO Clinical stage				< 0.001		
1	264	25 (9.5)	1-		1-	
2	99	18 (18.2)	2.1 (1.1–4.1)	0.02	1.7 (0.8–3.6)	0.07
3	138	11 (8.0)	0.8 (0.4–1.7)	0.6	0.7 (0.3–1.7)	0.49
4	9	5 (55.6)	11.9 (3.0–47.3)	< 0.001	8.8 (1.8–43.0)	0.007
CD4 count				0.002		
500 et Plus	158	10 (6.3)	1-			
200–500	292	35 (12.0)	2.0 (0.97–4.2)	0.06		
0–199	60	14 (23.3)	4.5 (1.9–10.8)	0.001		
Initial ART regimen						
TDF + 3TC + EFV	443	49 (11.1)	1-		–	
Other regimen	67	10 (14.9)	1.4 (0.7–2.9)	0.4		
Previous VL test				< 0.001		
< 20 copies/ml	343	19 (5.5)	1-		1-	
20–1000 copies/ml	49	7 (14.3)	2.8 (1.1–7.1)	0.03	2.4 (0.9–6.3)	0.07
> 1000 copies/ml	75	25 (33.3)	8.5 (4.4–16.6)	0.000	7.2 (3.5–14.5)	< 0.001
No previous VL test	43	8 (18.6)	3.9 (1.6–9.5)	0.002	3.1 (1.2–8.1)	0.02

ART: antiretroviral therapy; km: kilometer; ml: milliliter; N: number; OR: odds ratio; PIT: provider initiated testing; PMTCT: prevention mother to child transmission; VCT: voluntary counselling and testing; VL: viral load; WHO: World Health Organization

**Fig. 3:**
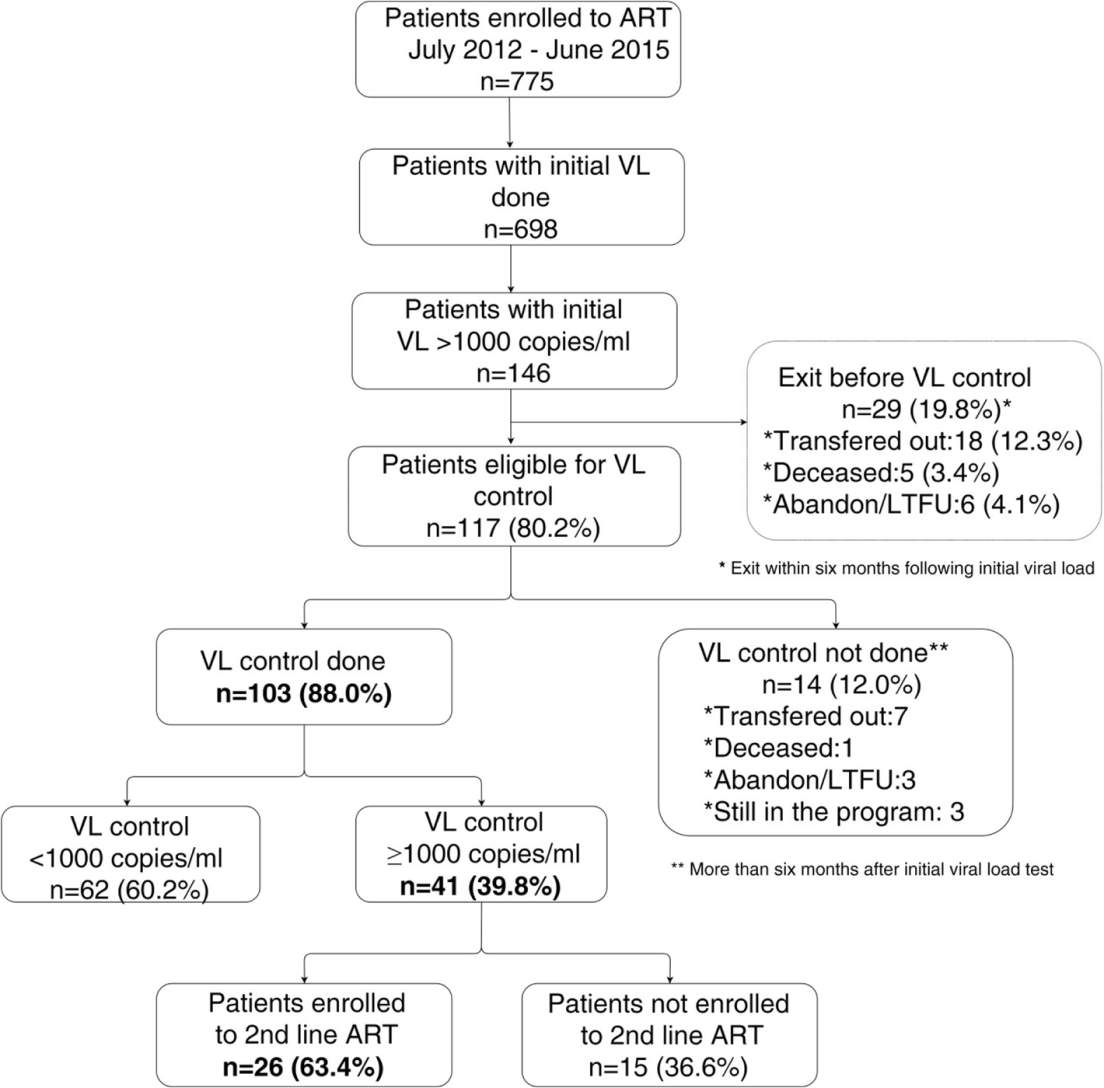
Management of patients with a viral load ≥1000 copies/ml in Rwanda, in Ruhengeri hospital and Musanze health center, 2013–2016. ART: antiretroviral therapy; VL: Viral Load; LTFU: lost to follow-up; n: number.

Considering all 775 patients enrolled on ART, 90% (*n* = 698) had an initial VL (first VL between July 2012 and June 2015), of which 146 (20.9%) patients had a high initial VL (≥ 1000 copies/ml). Out of 146 patients with a detectable VL, 29 exited the program without having a follow-up VL: 18 were transferred out, five died, and six were lost to follow-up. Among the 117 that were active 6 months after their high initial VL, 88.0% (*n* = 103) had a follow-up VL, of whom 60.2% (*n* = 62) had a VL below 1000 copies/ml (Fig. 3). Overall, the median time between the high initial VL and the follow-up VL was 12.5 months (IQR: 8.7–19.0). Among 41 patients with a follow-up VL higher than 1000 copies/ml, 26 (63.4%) started second-line ART (Fig. 3). The median time between the date of the follow-up VL and the date for second-line ART initiation was 17 days (IQR: 8–42).

## Discussion

This study from a rural district in Rwanda shows a high uptake of routine VL testing: 93.2% of eligible patient had a VL in 2016. Virological suppression was close to the 90% global UNAIDS target. Identified predictors of having a detectable VL were male gender, being a sex worker, having a WHO clinical stage IV when starting ART, having had a detectable VL before 2016, and having had no VL before 2016. The vast majority (88.5%) of patients with a high initial VL had a follow-up VL done. The median time between the initial and follow-up VL was 12.5 months. After a high initial VL, most (60.2%) patients had a suppressed follow-up VL. Most of patients (63.4%) with virologic failure (with a high follow-up VL) were initiated on second line ART.

The uptake of VL in rural area of Rwanda was remarkably high when compared to other sub-Saharan Africa studies [4]. Studies from Mozambique and Swaziland reported respectively 40 and 73% VL uptake [14, 15]. In countries where uptake was low, reported barriers included non-compliance with the WHO guideline regarding routine VL testing, lack of awareness among clinicians and patients on the benefits of VL testing, logistical constraints associated with sample transport, the long turnaround time between sampling and having a result available in the patient file, lack of funding for the implementation of routine VL testing, and the high cost of consumables required for VL testing [4, 10]. The high uptake of routine VL testing in our rural area of study, can be explained by a combination of factors, including increased accessibility after decentralization of the VL testing platform, training and sensitization of medical staff involved in HIV care, and gratuity of VL testing.

Virological suppression in patients on ART (88.5%) was lower than what was reported in patients on ART in Botswana (95.6%) and Malawi (90.8%) but higher than in most other settings [4, 14, 16, 17]. A close follow-up of groups at risk of having a detectable VL (males, sex workers, patients with WHO stage IV at ART initiation, and patients with previous detectable VL) would likely increase the number of patients on ART with virological suppression and contribute to achieve the third 90% UNAIDS target.

In patients with detectable VL, the vast majority (88%) had a follow-up VL done. The median time between a detectable VL and a follow-up VL was 12.5 months (IQR: 8.7–19.0). This period was twice as long as the period recommended by the national HIV guideline. Moreover, this contrasts with what was reported in South Africa, where a follow-up VL result was available after a median time of 4.9 months (IQR:2.8–6.1) [18]. Sessions of adherence counseling conducted on an extended period and the high volume of patients followed in HIV clinics may explain the long time observed between initial and follow up viral load. This resulted in a delay to switch some patients in need to the second-line ART. Considering that a delay to switch to the second-line ART is associated with a high risk of second-line virologic failure and an increased risk of death, [19, 20], patients failing the first line regimen would much benefit from measures to speed-up the switch to second-line ART.

Among patients with a high initial VL, 60.2% suppressed the follow-up VL after adherence counseling and did not require any modification of their first-line ART regimen. Likely, enhanced adherence counselling helped patients to improve their adherence to treatment. This finding illustrates the benefit of a routine VL monitoring, as it allows to identify patients in need of a close follow up, and to monitor their adherence. Indeed, previous studies have shown that targeted adherence support can help patients to re-suppress their detectable VL and keep taking the first-line ART regimen [21, 22].

In our study, almost two-third of our patients (63.4%) failing the first line ART were switched to the second-line ART before the end of the study period. This is quite similar to what was reported by a systematic review from 16 sub-Sahara African countries, which showed that 58% of patients with confirmed virologic failure switched to second-line ART [23]. Reported reasons for this low rate of switching to second-line ART include the desire of health care providers to optimize adherence counseling before second-line ART initiation, and deferrals of second line ART switch when patients miss scheduled appointments. [19, 20, 23] On the other hand, in our study, the decision to start second-line ART was generally taken quickly, within one month period following a confirmation of virologic failure.

The strengths of our study include that findings represent the real-life setting of an HIV program in rural Rwanda, as data were retrieved from routinely-collected patient-level program data. Moreover, we believe the findings are based on robust data. Data were retrieved from a prospectively updated electronic database, used for program monitoring. For the aim of this study, data were cleaned by comparing them with data from the individual patient files. No or very few values were lacking for most variables. Moreover, the vast majority of patients had effectively a VL done, which allowed us to evaluate the third UNAIDS target. However, there are some limitations as well. The two study sites, Ruhengeri hospital and Musanze health center are two neighboring health facilities, both located in the rural District of Musanze in the Northern Province of Rwanda. As there were no geographical barriers between the health facility where clinical care of patients was conducted and the site where VL testing was performed, the findings may not be generalizable to patients attending remote health facilities. Nevertheless, the findings show that when geographical barriers are minimized, routine VL monitoring is feasible to monitor the implementation of the ambitious UNAIDS 90–90-90 targets. Another limitation of the presented study is that adherence in terms of daily pill intake was not assessed. Moreover, the outcome of patients lost to follow-up was not known. As patients lost to follow up count generally among patients with low level of adherence on ART, they are more likely to have unsuppressed VL. It was also not possible to know the outcome of viral load testing for patients transferred out all along our study period.

## Conclusion

The uptake of routine VL monitoring was very high after decentralization of a VL testing platform in a rural area of Rwanda. The vast majority (88.5%) of patients tested had a suppressed VL. Among patients with detectable VL (≥1000 copies/ml), the majority of them re-suppressed after sessions of adherence counseling. Still, a close clinical follow-up of patients at risk of a detectable VL, such as patients with a previous detectable VL, patients who had a low CD4 count or advanced stage of disease when they started ART, female sex workers, may result in an earlier identification of virological failure. To shorten the time between a detectable and a follow-up VL, it is recommended an individualized patient monitoring and enhanced adherence counselling for patients with detectable VL. This may contribute to achieving and sustaining the third 90 UNAIDS target, aiming at having more than 90% of patients on ART with undetectable viral load.

## Abbreviations

3TC: Lamivudine
ART: Anti-retroviral therapy
EFV: Effavirenz
IQR: Interquartile Range
LTFU: Lost to follow up
PIT: Provider Initiated Testing
PLHIV: People living with HIV
PMTCT: Prevention of Mother to Child Transmission
RBC: Rwanda Biomedical Center
TDF: Tenofovir
UNAIDS: Joint United Nations Program on HIV/AIDS
VCT: Voluntary Counseling and Testing
VL: Viral load
WHO: World Health Organization
